# Evaluation of selenite reduction under salinity and sulfate stress in anaerobic membrane bioreactor

**DOI:** 10.3389/fbioe.2023.1133613

**Published:** 2023-03-10

**Authors:** Yuanyuan Zhang, Shuang Liu, Gaorong Zhang, Yixiang Peng, Qiaoyan Wei, Minmin Jiang, Junjian Zheng

**Affiliations:** ^1^ College of Life and Environmental Science, Guilin University of Electronic Technology, Guilin, China; ^2^ Guangxi Key Laboratory of Automatic Detecting Technology and Instruments, Guilin University of Electronic Technology, Guilin, China

**Keywords:** anaerobic membrane bioreactor (AnMBR), microbial reduction, selenite (SeO_3_^2−^), elemental selenium (Se^0^), sulfate (SO_4_^2−^), salinity

## Abstract

Current microbial reduction technologies have been proven to be suitable for decontaminating industrial wastewaters containing high concentrations of selenium (Se) oxyanions, however, their application is strictly limited by the elemental Se (Se^0^) accumulation in the system effluents. In this work, a continuous-flow anaerobic membrane bioreactor (AnMBR) was employed for the first time to treat synthetic wastewater containing 0.2 mM soluble selenite (SeO_3_^2−^). The SeO_3_^2−^ removal efficiency by the AnMBR was approachable to 100% in most of the time, regardless of the fluctuation in influent salinity and sulfate (SO_4_^2−^) stress. Se^0^ particles were always undetectable in the system effluents, owing to their interception by the surface micropores and adhering cake layer of membranes. High salt stress led to the aggravated membrane fouling and diminished content ratio of protein to polysaccharide in the cake layer-contained microbial products. The results of physicochemical characterization suggested that the sludge-attached Se^0^ particles presented either sphere- or rod-like morphology, hexagonal crystalline structure and were entrapped by the organic capping layer. According to the microbial community analysis, increasing influent salinity led to the diminished population of non-halotolerant Se-reducer (*Acinetobacter*) and increased abundance of halotolerant sulfate reducing bacteria (*Desulfomicrobium*). In the absence of *Acinetobacter*, the efficient SeO_3_^2−^ abatement performance of the system could still be maintained, as a result of the abiotic reaction between SeO_3_^2−^ and S^2-^ generated by *Desulfomicrobium*, which then gave rise to the production of Se^0^ and S^0^.

## 1 Introduction

Selenium (Se) contamination in aquatic ecosystems, arising mainly from the wastewater discharge of mining, refinery, and power production industries, has become a global environmental concern in recent years (Li et al., 2022). A chronic aquatic life criterion of 0.005 mg Se/L has been set by United States Environmental Protection Agency (USEPA), while the Se contents of typical industrial effluents could amount to 0.1–20 mg/L (Santos et al., 2015; Ruj et al., 2022). The environmental risks of Se were frequently correlated to the aquatic accumulation of inorganic Se oxyanions (with the toxicity of around 40 times greater than organic Se forms), in which the selenite (SeO_3_^2−^) is more pervasive and toxic over selenate (SeO_4_^2−^) (Santos et al., 2015). The carcinogenesis, cytotoxicity and genotoxicity effects of Se oxyanions exposure on aquatic living organisms and humans have been well-documented in the literature (Sun et al., 2014; Tan et al., 2016). Hence, Se oxyanions removal from contaminated waters is essential to eliminate associated environmental impacts.

Currently, the application of physiochemical approaches for Se oxyanions removal, e.g., adsorption, coagulation/flocculation and catalytic reduction, is limited by their high costs and requirements of post-treatment procedures (Tan et al., 2016; Ruj et al., 2022). In comparison, the biological method, based on anaerobic microbial reduction, may be a promising alternative due to its good adaptability to complicated wastewater quality, cost-efficient and eco-friendly nature (Nancharaiah et al., 2018). The soluble and toxic Se oxyanions can be effectively converted by specialized anaerobes to insoluble (colloidal) and less toxic elemental Se (Se^0^) nanoparticles (Li et al., 2022). Despite the recent advances in the anaerobic microbial treatment of Se-laden wastewaters, the scale-up application of this technology is significantly challenged by the suspension of massive Se^0^ nanoparticles in effluents of conventional anaerobic bioreactors (Jain et al., 2015; Staicu et al., 2015; Nancharaiah et al., 2018). The accumulation of Se^0^ nanoparticles can not only give rise to the failure of effluent quality to meet the corresponding maximal contamination levels (typically 0.01–0.05 mg Se/L), but possibly trigger secondary pollution because of the reoxidation of Se^0^ nanoparticles back to Se oxyanions in the oxygenated aquatic environments (Tan et al., 2016).

Anaerobic membrane bioreactor (AnMBR), which combines membrane filtration and microbial reduction processes, has obtained increasing attention for wastewater treatment in the past decades (Shahid et al., 2020). Compared to conventional activated sludge systems, the greater biomass concentration and longer sludge retention time (SRT) could be maintained in AnMBRs, thus enabling the small footprint and increased effluent quality of reactors as well as less production of residual sludge (Han et al., 2015; Zhang et al., 2021). We suppose that if applied for purification of Se-contaminated waters, in addition to the appreciable removal of Se oxyanions, the minimization of effluent Se^0^ accumulation might be realized in AnMBR, since the microbiologically produced Se^0^ nanoparticles were reported to have sizes ranging from 50 to 500 nm (Kamnev et al., 2017; Borah et al., 2021), which could possibly be intercepted by the surface micropores of membranes (typically micro- and ultra-filtration membranes) and/or membrane surface-attached cake layer. Hitherto, however, the feasibility of applying AnMBR for Se oxyanions removal has not yet been evaluated.

In this study, a lab-scale AnMBR equipped with immersed flat-sheet microfiltration membrane modules was constructed to treat synthetic SeO_3_^2−^-containing wastewaters. In particular, sulfate (SO_4_^2−^) and NaCl were introduced to the influent, on account of the fact that real Se-laden industrial wastewaters universally contain large amounts of SO_4_^2−^ and high salinity (Tan et al., 2016; Zhang et al., 2019). The variations in the contaminants (i.e., SeO_3_^2−^, SO_4_^2−^ and total organic carbon (TOC)) abatement and membrane fouling behaviors of AnMBR with changing influent SO_4_^2−^ concentration and salinity were investigated. The constituents of microbial products as well as morphologies and elemental compositions of crystal particles in the cake layers (formed at diverse operating phases on the membrane surface) were determined. The morphologies, elemental and mineralogical compositions of biogenic Se^0^ particles as well as surface functional groups of capping layer in the sludges were compared in the absence and presence of SO_4_^2−^ in the influent. The evolution of microbial community structure with changing influent composition was revealed.

## 2 Materials and methods

### 2.1 Reactor set-up

**FIGURE 1:**
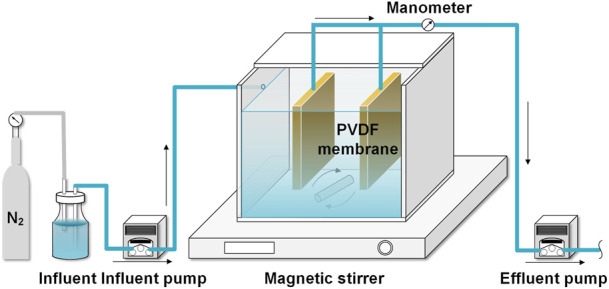
Schematic of the AnMBR.

As shown in Figure 1, a lab-scale AnMBR with an effective volume of 3.06 L (length × width × height = 15 × 12 × 17 cm) was constructed, in which 2 sheets of PVDF membrane modules (total filtration area = 0.048 m^2^, average pore size = 0.1 μm) were installed in the middle of the reactor. The AnMBR was inoculated by anaerobic sludge obtained from a WWTP in Guilin, Guangxi, China. A magnetic stirrer was employed to sustain the complete solid-liquid mixing in the reactor. The synthetic influent was pumped into the reactor after degassing by N_2_ for 10 min to eliminate the dissolved oxygen. The influent temperature was stabilized at 28°C ± 2°C (Pearce et al., 2009). Effluent flowrate of 2.125 ml/min was controlled by a peristaltic pump (BT101L, LeadFluid, China), which resulted in a hydraulic retention time (HRT) of 24 h, corresponding to the membrane flux of 2.66 LMH. The SRT was set at 60 days, and the concentration of volatile suspended solids (MLVSS) was maintained at around 4.2 g/L throughout the experiment. The membrane modules were operated at intermittent filtration mode with a suction/suspended time ratio of 10/2 min, and the transmembrane pressure (TMP) was monitored by an electronic pressure gauge. Once the TMP reached 30 kPa, the membrane was soaked in 0.5% (v/v) sodium hypochlorite for 2 h (Zheng et al., 2019). The reactor was operated at room temperature.

### 2.2 Experimental procedure

In accordance with previous studies (Kashiwa et al., 2000; Soda et al., 2011), the synthetic wastewater was prepared by tap water with the addition of 0.2 mM Na_2_SeO_3_, 0 or 8.4 mM Na_2_SO_4_, 7 mM NH_4_Cl, 0.17 mM K_2_HPO_4_, 0.07 mM KH_2_PO_4_, 0.2 mM MgCl_2_.6H_2_O, 0.2 mM CaCl_2_.2H_2_O, and 1 ml/L trace element stock. The tested SeO_3_^2−^ and SO_4_^2−^ concentrations are in their reported concentration ranges of 0–0.2 mM and 5–72 mM, respectively, in typical Se-laden industrial wastewaters (Tan et al., 2018). Sodium lactate of 12 mM was supplemented as the sole carbon source, and the resultant TOC was 432 mg/L. Influent salinity correlates to the application potential of AnMBR for treating Se-containing wastewater, since it can largely determine system performance by simultaneously affecting the microbial community and membrane fouling propensity (Jang et al., 2013; Luo et al., 2015). It has been documented that the excessively high influent salinity was able to inhibit the growth and SeO_3_^2−^ reduction of Se-reducer *Alteromonas* (Reddy et al., 2023). The salinity of typical Se-laden industrial wastewaters after preconditioning was reported to be around 1%, corresponding to a NaCl concentration of 10 g/L (Soda et al., 2011). Therefore, the salinity of influent was adjusted by adding 0–10 g/L NaCl. In line with this, NaCl was frequently employed as the representative of salinity when previous researchers attempted to evaluate the effects of salinity on bioreactor performance (Chen et al., 2019; Guo et al., 2020). The trace element stock was consisted of FeCl_2_.4H_2_O (0.05 mg/L), MnCl_2_.4H_2_O (0.15 mg/L), ZnCl_2_ (0.05 mg/L), CuCl_2_ (0.05 mg/L), CoCl_2_.6H2O (0.05 mg/L), and NiCl_2_.6H_2_O (0.05 mg/L). The influent pH was fixed at 7.0 ± 0.1 by the addition of 50% NaOH.

The AnMBR was operated over 4 phases as summarized in Table 1. During phase I, SO_4_^2−^ and NaCl were not incorporated in the influent. 5 g/L NaCl was added in the influent of phases II and III to acclimate halophilic Se-reducers, while to compare the system performance in the absence and presence of SO_4_^2−^, 806 mg/L SO_4_^2−^ was only supplemented in the influent of phase III. To evaluate the reactor performance under high salt stress, the influent NaCl concentration was further increased to 10 g/L during phase IV.

**TABLE 1:** Operational phases of AnMBR.

Operational phase	SeO_3_^2−^ (mg Se/L)	SO_4_^2−^ (mg/L)	TOC (mg/L)	NaCl (g/L)
Phase I (day 1–18)	15.8	0	432	0
Phase II (day 19–33)	15.8	0	432	5
Phase III (day 34–51)	15.8	806	432	5
Phase IV (day 52–73)	15.8	806	432	10

### 2.3 Microbial community analysis

The sludge samples, including the inoculum and sludges collected from the reactor at the end of each phase, were delivered to Sangon Biotech Co., Ltd. (Shanghai, China) for high-throughput sequencing analysis. The extraction of total genomic DNA in the samples was processed by E. Z.N.A^™^ Mag-Bind Soil DNA Kit (M5635-02, Omega, United States), according to the protocol described in the manufacturer’s instruction. Primer pairs of 341F (5′-CCTACGGGNGGCWGCAG-3′) and 805R (5′-GACTACHVGGGTATCTAATCC-3′) were selected for the amplification of the V3-V4 regions of bacterial 16 S rRNA genes, and have been widely applied to amplify bacteria under salinity stress (Herlemann et al., 2011). The amplified PCR products experienced purification, and then sequencing was performed using the Illumina MiSeq system (Illumina MiSeq, United States). Effective tags were assigned into operational taxonomic units (OTUs) with a similarity threshold of 97% using Usearch (V 11.0.667), and the tag sequence with the greatest abundance was designated as the representative sequence within each cluster. Diversity indices were calculated by Mothur (V 3.8.31) in terms of OTU richness. The sequencing data has been deposited in GenBank with accession number of SUB12506422.

### 2.4 Analytical methods

The concentration of MLVSS was measured according to APHA standard methods (APHA, 1995). Influent and effluent of the reactor were daily collected. After centrifugation (8000 × *g*, 10 min, 4°C) and filtration through 0.22 µm membrane filter, soluble SeO_3_^2−^, SO_4_^2−^, and TOC concentrations in supernatant were determined. Solid Se in the pellet was digested as described previously (Zhang et al., 2019). Se and TOC concentrations were measured by inductively coupled plasma atomic emission spectroscopy (ICAP RQ, Thermo Scientific, United States) and total organic carbon analyzer (TOC-L, Shimadzu, Japan), respectively. SO_4_^2−^ concentrations were detected on ion exchange chromatography (HIC-20ASP, Shimadzu, Japan). Soluble microbial products (SMP) and extracellular polymeric substances (EPS) were extracted according to the literature (Han et al., 2015). Polysaccharide (PS) and protein (PN) were determined by the anthrone-sulfuric acid and Lowry Folin methods, respectively. Morphologic characteristics and elemental composition of the Se^0^-coated sludges and membrane foulants obtained at the end of phases II-IV were inspected on scanning electron microscopy (SEM, Quanta 450 FEG, FEI, United States) coupled with energy dispersive X-ray spectroscopy (EDS, X-Max20, Oxford, England). The freezing dried biomass at the end of phases II and III were subjected to X-ray diffraction (XRD) analysis (SmartLab SE, Rigaku, Japan). The surface functional groups of inoculum and sludge samples were characterized by Fourier-transform infrared spectroscopy (FTIR, Niolet iN10, Thermo Scientific, United States).

## 3 Results and discussion

### 3.1 Reactor performance

**FIGURE 2:**
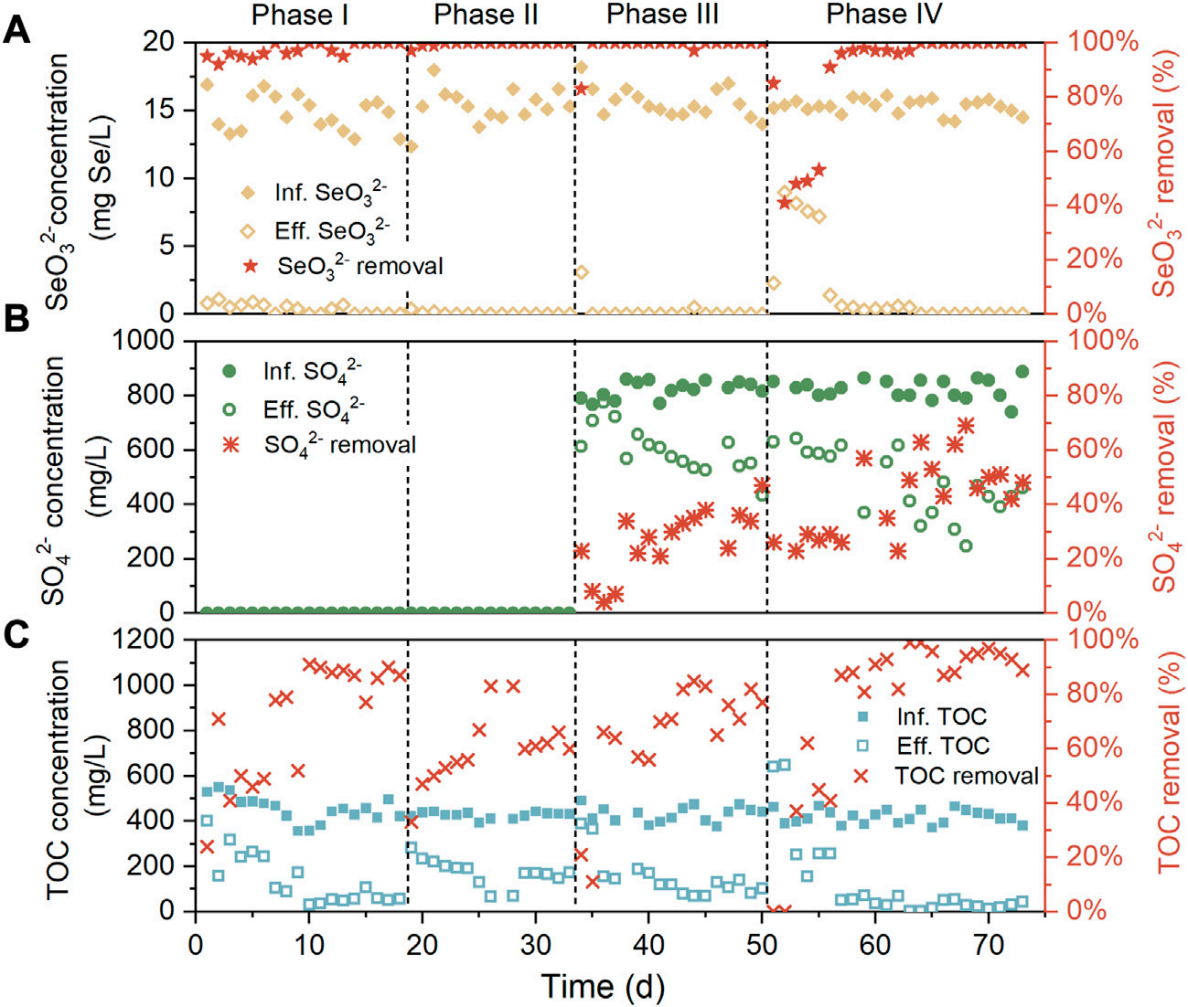
Removal performance of AnMBR for **(A)** SeO_3_^2−^ **(B)** SO_4_^2−^, and **(C)** TOC.

Removal performance of SeO_3_^2−^, SO_4_^2−^, and TOC over the four phases was evaluated with the results presented in Figures 2A–C. In the absence of SO_4_^2−^ and NaCl in the influent during phase I, the inoculum was capable of efficiently biotransforming SeO_3_^2−^ from the initial, resulting in about 95% of SeO_3_^2−^ removal on the first day, and from day 14, the complete SeO_3_^2−^ removal was achieved. This implied the rapid enrichment or selection of Se-reducers to the reactor environment. Meanwhile, the TOC abatement of the system surged from below 30% on day 1 to over 80% after day 10, likely associated with the proliferation of heterotrophic Se-reducers. When 5 g/L NaCl was supplemented in the influent (phase II), the TOC removal experienced a significant drop but ultimately recovered to around 70%. A possible reason for this phenomenon is that the increased extracellular osmotic pressure resulted in the lysis of non-halotolerant heterotrophic bacteria, followed by the surge of other halotolerant species. After 806 mg/L SO_4_^2−^ was added in the influent during phase III, the SO_4_^2−^ removal first increased and then stabilized at 20%–40%, with this variation trend similar to that of TOC removal. This was perhaps due to the proliferation of heterotrophic sulfate reduction bacteria. During phases II and III, SeO_3_^2−^ was undetectable in most effluent samples, indicating the insignificant influence of moderate influent salinity and SO_4_^2−^ concentration on the SeO_3_^2−^ reduction efficiency of the system. In accordance with this, previous studies have suggested that SeO_3_^2−^ is more energetically favorable electron acceptor for Se-reducers than SO_4_^2−^, according to thermodynamic calculations (Nancharaiah and Lens, 2015); SO_4_^2−^ negatively impacted Se reduction only when its concentration was beyond 150-folds of the latter (Tan et al., 2018), while the influent concentration ratio of S to Se was merely 41:1 in this research. As the influent NaCl content was further increased to 10 g/L during phase IV, the declined SeO_3_^2−^ removal was observed in the first several days (day 52–63), then SeO_3_^2−^ removal subsequently raised to 100% from day 64. It was also found that the recovery of SeO_3_^2−^ removal was along with the increase of SO_4_^2−^ and TOC removal. This was possibly owing to the facilitated SeO_3_^2−^ and TOC abatement by the enrichment of heterotrophic and halotolerant sulfate reduction bacteria, which will be discussed later. Throughout the experiment, Se^0^ was always undetectable in the system effluents.

### 3.2 Membrane fouling propensity

**FIGURE 3:**
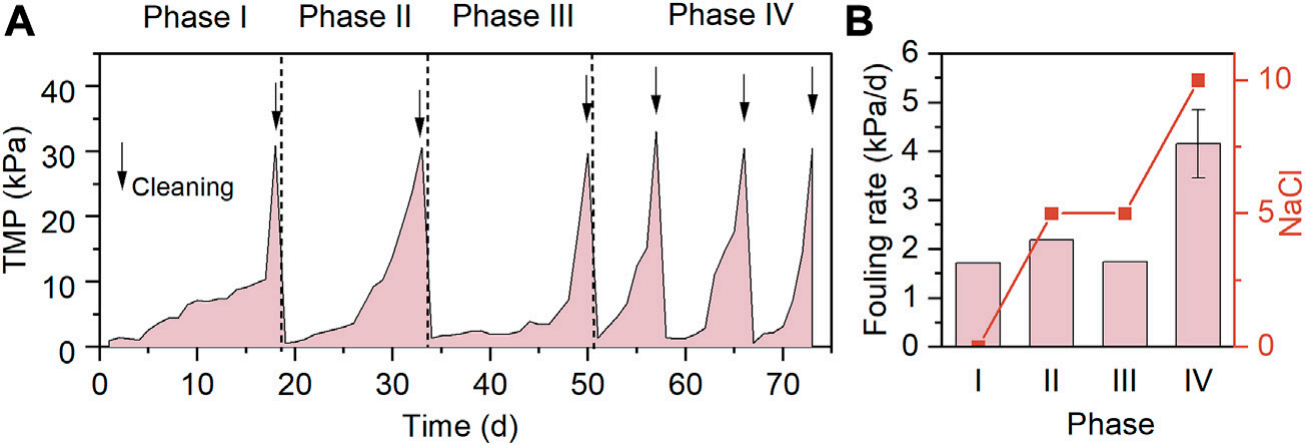
TMP profiles **(A)** and membrane fouling rates **(B)** of AnMBR.

The TMP profiles and membrane fouling rates of the system in the case of varying influent salinities and SO_4_^2−^ concentrations are presented in Figures 3A, B, respectively. Phases I-IV were all operated for at least 15 days to allow the microbial community structure to reach steady-state conditions at the end of each phase. In the absence of NaCl and SO_4_^2−^ in the influent during phase I, the TMP surpassed 30 kPa after 18 days operation, and the resultant fouling rate was 1.72 kPa/d. As 5 g/L NaCl was added in the influent, the fouling rate was slightly increased to 2.19 kPa/d (phase II), implying the negative influence of the increased influent salinity on the membrane filtration performance. The presence of SO_4_^2−^ in the influents of AnMBRs was found to exacerbate the membrane fouling by inducing the development of dense cake layer on the membrane surface (Oztemur et al., 2020; Zhou et al., 2020). However, it was found that the co-addition of 806 mg/L SO_4_^2−^ and 5 g/L NaCl during phase III resulted in a fouling rate of 1.74 kPa/d, lower than that at the phase II. A plausible explanation is that the release of organic cellular constituents (e.g., PN and PS) from the dead non-halotolerant heterotrophic bacteria during phase II accelerated the membrane fouling (Chen et al., 2019), while during phase III, the relatively slower membrane fouling was due to the better adaptability of the newly developed microbial community to the identical influent salinity. Further increasing the influent NaCl concentration to 10 g/L NaCl led to the greatest fouling rate of 4.16 kPa/d, and the membranes were subject to 3 times of chemical cleaning during phase IV. In line with this, the TMP of MBRs rapidly increased at high NaCl loadings (Jang et al., 2013; Luo et al., 2015).

**FIGURE 4:**
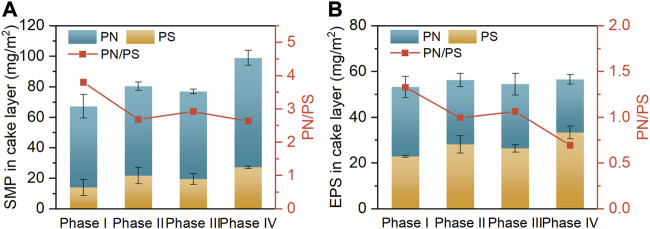
Contents of PN, PS and PN/PS ratios of in SMP **(A)** and EPS **(B)** of cake layers.

The aggravated membrane fouling incurred by high salt stress is known to be closely linked to the changed composition of biofilm (cake layer) (Wang et al., 2016). The contents of predominant components (i.e., PN and PS) in SMP and EPS of the cake layers collected at the end of diverse operating phases are depicted in Figures 4A, B, respectively. As the influent NaCl concentration was elevated from 0 g/L (phase I) to 10 g/L (phase IV), the SMP content of cake layer markedly increased from 67.3 to 99.0 mg/m^2^, accompanied by the marginal increase in its EPS content from 53.3 to 56.6 mg/m^2^. The significantly greater content of SMP over EPS was attributable to the fact that the solubilities of PN and PS augment with increasing influent salinity, thus enhancing the distribution of these fractions in the SMP (Zhang et al., 2014; Luo et al., 2015). Notably, from phase I to IV, the diminished PN/PS ratios were simultaneously found in the SMP (from 3.8 to 2.6) and EPS (from 1.3 to 0.7). These results are in good agreement with the findings of previous studies that the microorganisms in the activated sludges tended to secrete more PS to protect them against the cellular damage caused by salt shock, since PS could alleviate the dehydration of microbial cells by restricting water transport (Wang et al., 2016; Guo et al., 2020). Nevertheless, the increased content of PS could promote the generation of sticky hydrogels on membrane surface, thereby accelerating the membrane fouling (Jang et al., 2013). The increased PN/PS ratios in the biofilm-contained SMP and EPS at phase III than phase II were ascribed to the evolution of microbial community, as mentioned earlier.

**FIGURE 5:**
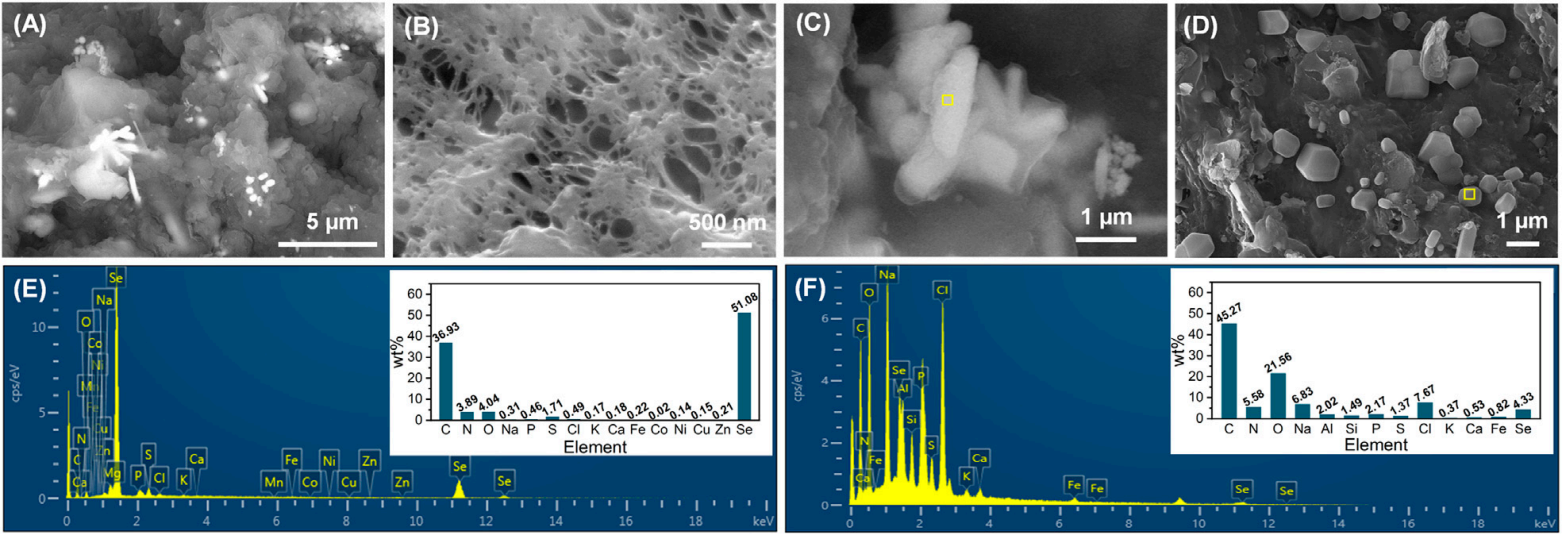
SEM images of cake layer **(A)** and membrane surface (after the stripping of cake layer and rinsing by deionized water) **(B)** at the end of phase IV, rod-like **(C)** and cube-shaped **(D)** crystal particles on the cake layer; EDS spectra of rod-like **(E)** and cube-shaped **(F)** crystal particles, corresponding to the specific area (marked in yellow) in their SEM images.

At the end of phase IV, the cake layer was stripped from the membrane modules and the membrane was surface-rinsed by deionized water, followed by their surface morphological analysis. As exhibited in Figures 5A, B, crystal particles were found to be largely dispersed on the cake layer, but did not appear in the membrane pores. The high magnification SEM images, as shown in Figures 5C, D, demonstrate that the crystal particles presented the rod-like and cube-shaped morphologies. The rods with an average dimension of 2000 × 500 nm were further corroborated to be Se^0^ particles, and the cubes were identified as NaCl crystals by EDS analysis (Figures 5E, F). The appearance of NaCl crystals in the membrane foulants has also been observed in a previous study where an influent NaCl concentration higher than 10 g/L was employed (Cai et al., 2021). The biogenic Se^0^ particles were reported to have typical sizes of below 500 nm (Kamnev et al., 2017; Borah et al., 2021), smaller than that observed in this study. The presence of large Se^0^ particles in the activated sludge could not only assure the good settleability of sludge (Zhang et al., 2019), but also benefit their subsequent extraction from the sludge. More importantly, the clean membranes were capable of intercepting Se^0^ particles *via* the size-exclusion effect, and upon the occurrence of membrane fouling, the formed cake layer could also contribute to the entrapment of Se^0^ particles. This advantage of AnMBR would enable it as a more attractive candidate over conventional anaerobic activated sludge systems for Se oxyanions removal.

### 3.3 Characteristics of sludge-attached Se^0^ particles

**FIGURE 6:**
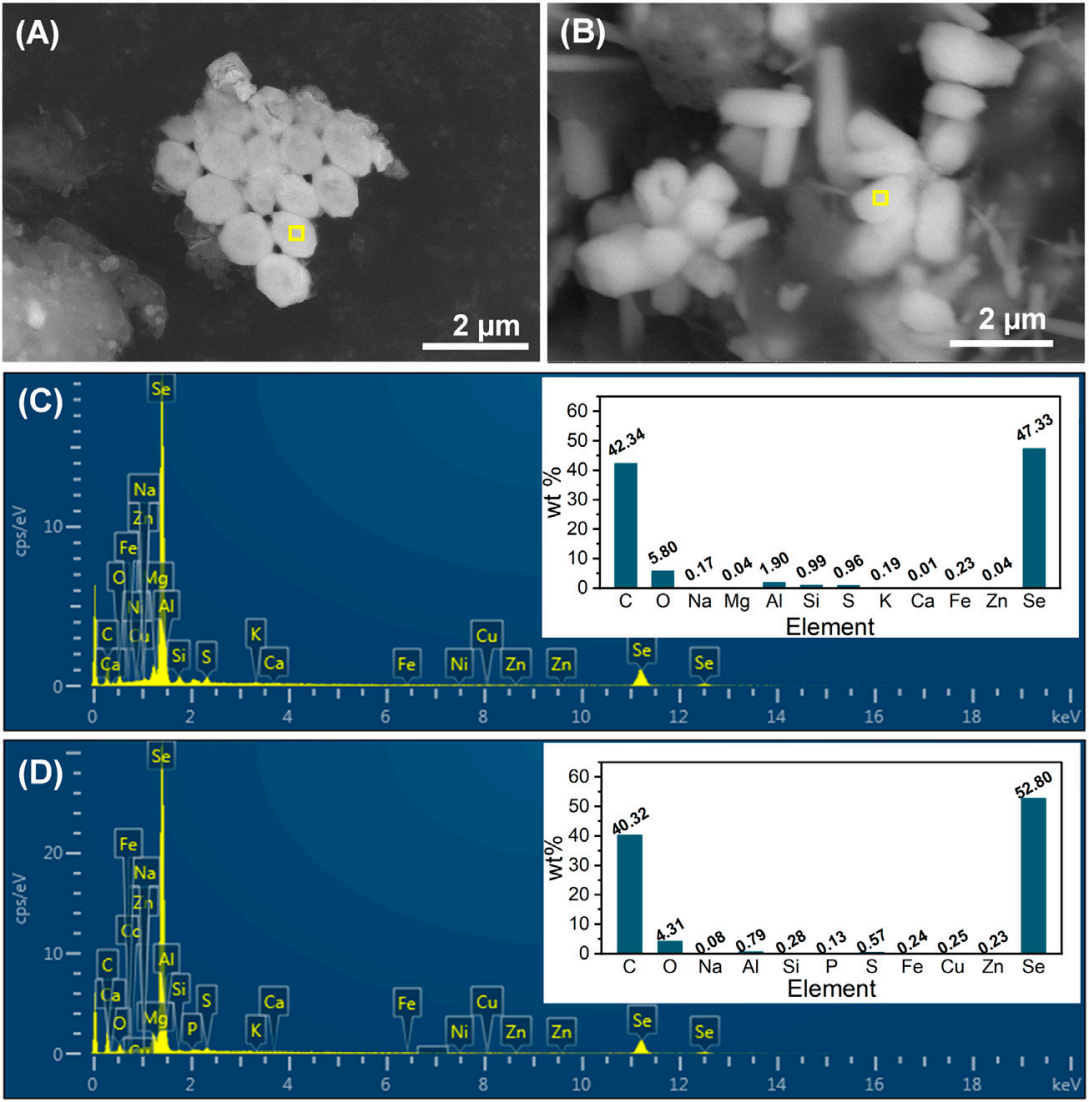
SEM images and EDS spectra (corresponding to the yellow-marked area of SEM images) of sludge-attached Se^0^ particles obtained at the end of phases II **(A, C)** and III **(B, D)**.

Sludge-attached Se^0^ particles obtained at the end of phases II and III were characterized by SEM-EDS (Figure 6A–D). From the combination analysis of Figures 5C, E and Figure 6A–D, it can be found that the elemental proportions of Se in all Se^0^ particles were similar (47.33%–52.80%), but in contrast to the rod-like morphology of Se^0^ particles at phases III and IV, the Se^0^ particles obtained at phase II exhibited the sphere-like feature with an average of around 800 nm. The morphological change of Se^0^ particles was possibly associated with their growth with the extension in the operating time of system. In the literature, the microbiologically produced Se^0^ particles in batch reactors presented the sphere-like morphology with nano sizes (Jain et al., 2015; Borah et al., 2021), but were inclined to appear as rods with relatively larger size (up to 10 μm in length) in the long-term operated reactor (Zhang et al., 2019). The appearance of other elements with high elemental proportions, i.e., C, O and N, can be owing to the presence of organic capping layer on the surface of Se^0^ particles, which were deemed as primarily EPS-contained PN, PS and lipids (Borah et al., 2021).

**FIGURE 7:**
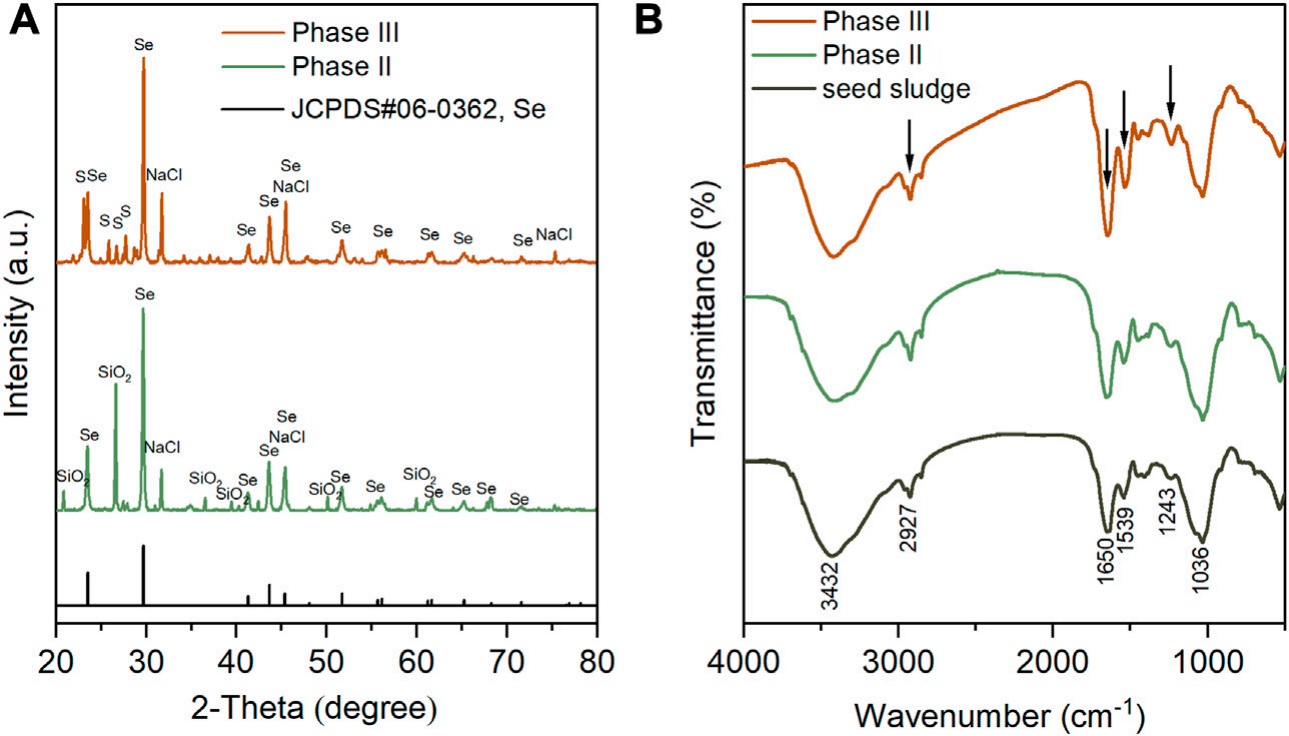
XRD **(A)** and FTIR **(B)** spectra of sludge-attached Se^0^ particles collected at the end of phases II and III. In particular, seed sludge was used as control group of the FTIR spectra.

The phase composition of sludge-attached Se^0^ particles collected at the end of phases II and III was analyzed. As exhibited in Figure 7A, in the XRD spectra of two samples, the appearance of multiple characteristic diffraction peaks, which corresponded perfectly to standard XRD card JCPDS#06–0363, indicating the hexagonal crystalline structure of the generated Se^0^ particles. Compared to the amorphous Se^0^ form, hexagonal crystalline Se^0^ particles, which was considered to be originated from the allotropic transition of amorphous Se^0^ (Ruiz-Fresneda et al., 2023), is more thermodynamically stable (Wang et al., 2017; Nancharaiah et al., 2018; Liu et al., 2022; Song et al., 2022). This implies that the generated Se^0^ particles were less likely to be oxidized back to Se oxyanions to cause secondary pollution. The peaks at 31.7° (200), 45.5° (220), 75.3° (420) confirmed the presence of cubic NaCl (JCPDS#99–0059), in agreement with the SEM-EDS analysis results, as shown in Figure 5D, F. The NaCl crystallization was likely ascribed to its concentration polarization at the membrane-solution interface during the filtration process (Yang et al., 2002), and similar phenomenon had been observed by a previous study in which 10–35 g/L influent NaCl concentrations were adopted (Cai et al., 2021). The peaks at 20.9°, 26.7°, 36.6°, 39.6°, 50.2° and 60.0° in the XRD spectra of the sample obtained during phase II are possibly attributed to the introduction of SiO_2_ particles during its grinding preparation in a quartz mortar. Orthorhombic S^0^ (JCPDS#99–0066) was found to co-exist with Se^0^ in the sludge collected during phase III, by observation of the characteristic peaks at 23.1° (222), 25.8° (026), 26.7° (311) and 27.7° (206) in the corresponding XRD spectrum.

The functional groups of seed sludge and the sludges obtained at the end of phases II and III were determined by FTIR (Figure 7B). The broad peak at 3,432 cm^−1^ was assigned to hydroxyl groups (Sinharoy et al., 2019), and the other broad peak at 1,036 cm^−1^ was ascribed to the C–O groups in oligo and PS (Sinharoy and Lens, 2020). In comparison to the seed sludge, the markedly higher intensities of peaks at 2,927, 1,650, 1,539, and 1,243 cm^−1^ appeared in the FTIR spectra of the sludges collected after Se^0^ formation. The band at 2,927 cm^−1^ was associated with the stretching vibration of C–H in ν(CH_2_) (Kamnev et al., 2017). The enhancement in intensity of this band was attributed to the increased production of lipids (i.e., aliphatic chains of fatty acid) (Kamnev et al., 2017). The greater intensities of peaks at 1,650, 1,539, and 1,243 cm^−1^ were closely related to the increasing contents of amid I, amid II, and amid III in the side-chains of cellular PN, respectively (Kamnev et al., 2017). In accordance with the EDS analysis results in Figure 5E, F and Figure 6C, D, these findings suggested that the microorganisms in the sludges could secrete more microbial products (e.g., lipids and PNs) to form an organic capping layer to entrap the biogenic Se^0^ particles. It has been documented that the existence of organics (especially PN) on the surface of Se^0^ particles could prevent their agglomeration, and then hinder their transformation from amorphous to crystalline structure (Hageman et al., 2013; Hnain et al., 2013). In this study, only crystalline Se^0^ particles were determined, presumably associated with the relatively low PN content in the organic capping layer (Wadhwani et al., 2017). Despite the aforementioned findings, a quantitative investigation of the sludge- and cake layer-attached Se^0^ particles is still anticipated to illuminate their fate in the AnMBR.

### 3.4 Microbial community evolution

Based on the qualified sequence numbers, the sludge samples were classified into 1,774 OTUs (Table 2). Diversity indexes were calculated based on the output of OTUs. Both Shannon diversity and Chao1 richness showed a decreasing trend with the step-wise addition of SO_4_^2−^ and NaCl in the influent, likely due to the elimination of non-halotolerant bacteria and proliferation of halotolerant SO_4_^2−^ and/or SeO_3_^2−^ reducers.

**TABLE 2:** Summary of OTUs and diversities of microbial communities.

Operational phase	Qualified sequence number	OTUs	Shannon index	Chao1 estimator	Good’s coverage
Seed sludge	81,315	1,559	5.99	1,608	1
Phase I	74,837	1,210	2.63	1,450	1
Phase II	82,577	884	3.22	1,052	1
Phase III	95,448	443	2.94	660	1
Phase IV	105,447	177	2.57	231	1

**FIGURE 8:**
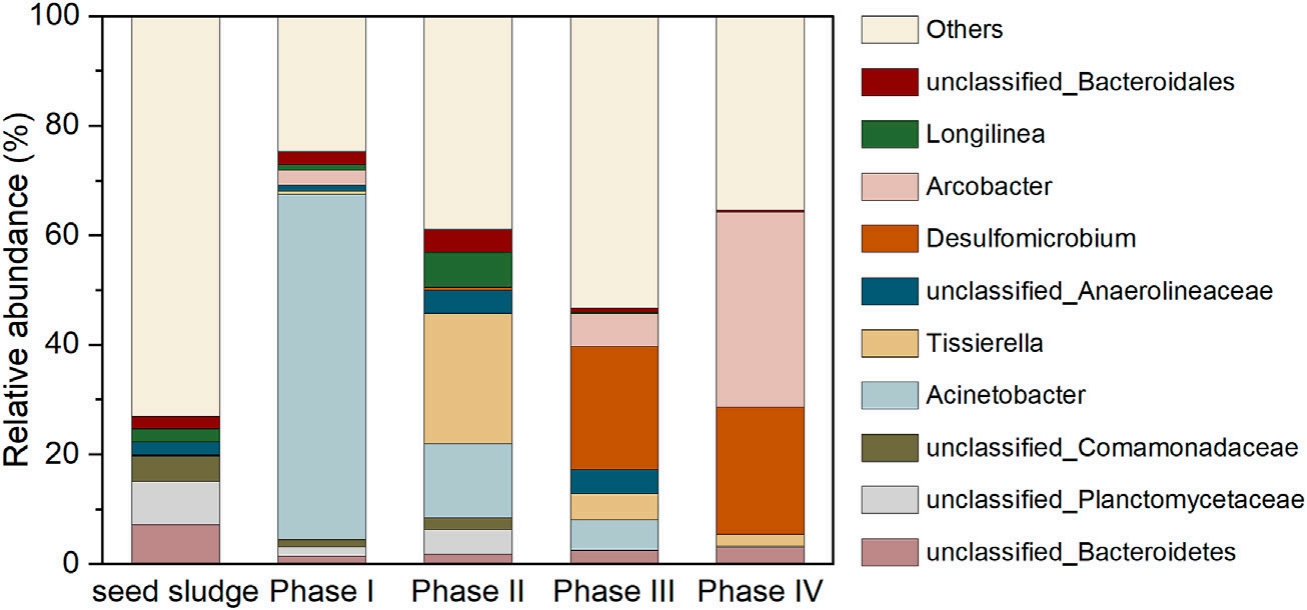
Dominant genera in microbial communities (top 10).

Figure 8 depicts the evolution of microbial community. The unidentified bacteria belong to phylum *Bacteroidetes* (7.24%), family *Planctomycetaceae* (7.91%) and family *Comamonadaceae* (4.58%) dominated in the seed sludge, which were not able to be designated as any genus with similarity of above 97%, had been frequently detected in anaerobic environments. The genus *Acinetobacter* (62.96%), affiliated to *Proteobacteria*, dramatically thrived as 0.2 mM SeO_3_^2−^ was added in the influent during phase I. The cell suspension as well as cell protein of this genus (*Acinetobacter* sp. sW30) was responsible for the conversion of SeO_3_^2−^ to Se^0^ particles, thereby contributed to the elimination of SeO_3_^2-^ in the AnMBR (Wadhwani et al., 2017). After the introduction of 5 g/L NaCl in the influent during phase II, the declined abundance of *Acinetobacter* was along with the increased population of *Tissierella* (23.72%). This variation likely resulted from the increased salt stress, because of the halotolerant advantage of *Tissierella* (Chen et al., 2020). The members of this genus utilize proteinaceous substrates for growth, which were probably originated from the autolysis of non-halotolerant microbes (Chen et al., 2020). The unclassified *Anaerolineaceae* and *Longilinea* belonged to the family of *Anaerolineaceae*. The dominant genera shifted to *Desulfomicrobium* (22.43%) when extra SO_4_^2−^ was supplemented in the influent (phase III). As one of the most universal sulfate reducing bacteria with the capacity to survive in saline environments, *Desulfomicrobium* was assumed to dominate the SO_4_^2−^ removal during phases III and IV (Dias et al., 2008; Guo et al., 2019; Ganesan et al., 2022). It is noteworthy that in terms of functional microbial population, the decreased Se-reducer (*Acinetobacter*) was accompanied by the increase of sulfate reducing bacteria (*Desulfomicrobium*) from phase I to IV. However, the almost complete SeO_3_^2−^ elimination was achieved during most time of all phases, as shown in Figure 2. This was because in the absence of Se-reducer, the S^2−^ generated by *Desulfomicrobium* could still abiotically react with SeO_3_^2−^ in vitro via redox reaction to generate the elemental Se^0^ and S^0^, thus maintaining the efficient SeO_3_^2−^ removal performance of the system (Hockin and Gadd, 2003; Pettine et al., 2012). This also helped explain the emergence of orthorhombic S^0^ in the XRD spectrum of sludge-attached Se^0^ particles collected at the end of phase III, as exhibited in Figure 7A. The SO_4_^2−^ is not likely a decisive factor influencing the generation of crystalline Se^0^ by Se-reducers, since it has been corroborated by previous studies that hexagonal crystalline Se^0^ could be generated by Se-reducers in the absence of SO_4_^2−^ (Jain et al., 2015; Nancharaiah et al., 2018). *Arcobacter*, a widely detected halotolerant sulfide oxidizer, was found to thrive during phases III and IV (Wirsen et al., 2002; Virpiranta, 2022). Other Se-reducer with minor relative abundances: *Pseudomonas* (0%–1.00%), *Shewanella* (0%–0.03%), *Rhodobacter* (0%–0.02%). Other sulfate-reducer: *Desulfuromonas* (0%–0.04%), *Desulfovibrio* (0.02%–0.36%).

Overall, in the absence of SO_4_^2−^ during phases I and II, the Se-reducer (mainly *Acinetobacter*) was responsible for the biotic conversion of SeO_3_^2−^ to Se^0^; after SO_4_^2−^ introduction in the influent during phase III, Se-reducer and sulfide produced by sulfate reducing bacteria (mainly *Desulfomicrobium*) contributed to the biotic and abiotic Se^0^ production, respectively; the abiotic SeO_3_^2−^ reduction accounted for the Se^0^ generation during phase IV, because high influent salinity led to the proliferation of sulfate reducing bacteria and disappearance of Se-reducer.

## 4 Conclusion

This study reported for the first time that the AnMBR could be a promising technology for efficient SeO_3_^2−^ removal from wastewater while preventing secondary pollution. The presence of high concentrations of SO_4_^2−^ and NaCl in the influent did not significantly suppress the SeO_3_^2−^ reduction process in the long-term operated system, despite the transient inhibition effect existed. Increasing influent salinity could accelerate the membrane fouling by inducing a declined ratio of cake layer-contained PN/PS. Se^0^ particles, featuring either sphere- or rod-like morphology, the hexagonal microstructure and entrapment by organic capping layer, were identified as the SeO_3_^2−^ reduction products, which were completely retained in the reactor. SeO_3_^2−^ reduction was dominated by Se-reducer when NaCl was not introduced in the influent, and might have been produced by abiotic redox reaction initiated by the sulfate reducing bacteria-produced S^2−^, in the case of high influent salinity.

## Data Availability

The datasets presented in this study can be found in online repositories. The names of the repository/repositories and accession number(s) can be found in the article/supplementary material.
